# Graphene-Based Electrodes for Silicon Heterojunction Solar Cell Technology

**DOI:** 10.3390/ma14174833

**Published:** 2021-08-26

**Authors:** Ignacio Torres, Susana Fernández, Montserrat Fernández-Vallejo, Israel Arnedo, José Javier Gandía

**Affiliations:** 1 Departamento de Energías Renovables, Centro de Investigaciones Energéticas, Medioambientales y Tecnológicas (CIEMAT), 28040 Madrid, Spain; jj.gandia@ciemat.es; 2 das-Nano, Polígono Industrial Talluntxe, Calle M-10, Tajonar, 31192 Navarra, Spain; iarnedo@das-nano.com (I.A.); mfernandez@das-nano.com (M.F.-V.); 3 Departamento Ingeniería Eléctrica, Electrónica y de Comunicación, Universidad Pública de Navarra, 19, Campus Arrosadía, 31006 Pamplona, Spain

**Keywords:** graphene, transparent conductive electrodes, ITO, solar cells, silicon heterojunction, terahertz time-domain spectroscopy

## Abstract

Transparent conductive electrodes based on graphene have been previously proposed as an attractive candidate for optoelectronic devices. While graphene alone lacks the antireflectance properties needed in many applications, it can still be coupled with traditional transparent conductive oxides, further enhancing their electrical performance. In this work, the effect of combining indium tin oxide with between one and three graphene monolayers as the top electrode in silicon heterojunction solar cells is analyzed. Prior to the metal grid deposition, the electrical conductance of the hybrid electrodes was evaluated through reflection-mode terahertz time-domain spectroscopy. The obtained conductance maps showed a clear electrical improvement with each additional graphene sheet. In the electrical characterization of the finished solar cells, this translated to a meaningful reduction in the series resistance and an increase in the devices’ fill factor. On the other hand, each additional sheet absorbs part of the incoming radiation, causing the short circuit current to simultaneously decrease. Consequently, additional graphene monolayers past the first one did not further enhance the efficiency of the reference cells. Ultimately, the increase obtained in the fill factor endorses graphene-based hybrid electrodes as a potential concept for improving solar cells’ efficiency in future novel designs.

## 1. Introduction

Silicon heterojunction (SHJ) solar-cell technology is securing a space in the large-volume manufacturing PV market, currently dominated by BSF cells, PERC and PERT cells, thanks to its impressive efficiency and relatively simple structure [1]. Essentially, the structure consists of a crystalline silicon wafer sandwiched between passivating selective contacts made of hydrogenated amorphous silicon (a-Si:H), intrinsic/n-doped and intrinsic/p-doped stacks. The excellent passivation properties of amorphous silicon allow SHJ solar cells to achieve very high open-circuit voltages (*V*_oc_) in excess of 740 mV. In addition, by employing amorphous silicon in extremely thin layers the parasitic absorption can be minimized and high short-circuit currents (*J*_sc_) can be obtained. However, the lateral electrical conductivity in these layers is insufficient for providing good carrier transport towards the metal-grid fingers, so a transparent conductive oxide (TCO) is needed. Typically, indium tin oxide (ITO) is the TCO of choice for SHJ technology with aluminum-doped zinc oxide also being widely studied as a potential substitute of ITO due to the scarcity of indium [2]. Since the optimization of the TCO is a trade-off between transparency and electrical conductivity, the TCO’s sheet resistance usually represents an important source of the solar-cell series resistance [3].

High transparency and high electrical conductivity are two characteristics typically associated with nanoscale materials, such as carbon nanotubes, graphene, metal nanowires and metal nanogrids. Hence, transparent conductive electrodes (TCE) based on nanomaterials have been integrated into a wide variety of electro-optical applications (LEDs, solar cells, touch-screens, etc.) with promising results [4]. However, in the case of SHJ solar cells, the TCO also serves as an antireflectance (AR) coating. Therefore, any substitute of ITO in SHJ technology needs to address this, otherwise the solar-cell efficiency can be highly hindered [5]. One way to benefit from nanomaterials’ exciting properties while utilizing AR characteristics is by fabricating hybrid electrodes. For example, hybrid structures based on ITO and graphene have already shown excellent electrical and optical properties [6,7] suitable for implementation in SHJ solar cells. In this paper, the concept of transparent hybrid electrodes based on ITO and graphene monolayers (GML) grown via chemical vapor deposition (CVD) is explored further with its application to SHJ solar cells.

The optical and electrical properties of hybrid electrodes based on ITO and GML can be modified through the number of GML used. In this work, hybrid electrodes with up to three GML are tested. The hybrid electrodes are fabricated directly in SHJ structures before metal grid deposition. Therefore, the results obtained from the characterization of the hybrid electrodes and the properties of the finished solar cells are closely related. The quality of the graphene layers was established through Raman measurements, whereas the AR properties of the hybrid electrodes was evaluated through reflectance spectroscopy. Contactless measurements of the electrodes sheet resistance were evaluated through THz time domain spectroscopy, illustrating how the hybrid electrodes exhibit a lower sheet resistance with an increased number of GML. Thanks to the lower sheet resistance, the finished solar cells with hybrid electrodes showed improvements in the series resistance and *FF* when compared with the reference cell with only ITO. On the other hand, by increasing the number of GML, the transmittance of the hybrid electrodes decreased accordingly and *J*_sc_ was negatively affected. However, by limiting the number of GML to only one, the gains in *FF* outmatched the losses in *J*_sc_ and the efficiency of the devices improved with respect the reference cell. These results highlight the potential for the development of advanced transparent or semitransparent electrodes based on graphene and its application to optoelectronic devices.

## 2. Materials and Methods

The goal of this research is to evaluate the effect of combining multiple layers of graphene with ITO as the front transparent conductive electrode in SHJ solar cells. For that purpose, very high-quality graphene films were used. In particular, we employed CVD grown graphene purchased from Graphenea S.A., a company specialized in the manufacturing and application of graphene (see [8] for further details). Furthermore, in order to assure a pristine transferring process, carried out in optimum conditions, the films were transferred in Graphenea’s own facilities.

**Figure 1:**
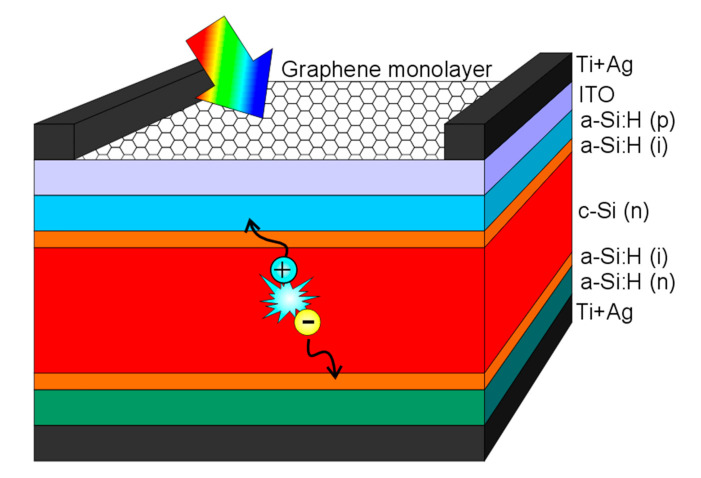
Sketch of the finished silicon heterojunction solar cell, in which the front ITO layer has been replaced with a graphene-based hybrid transparent conductive electrode consisting of an 80 nm-thick ITO layer and a stack of up to three graphene monolayers.

SHJ solar cells were fabricated using a 280 µm-thick flat n-type float zone c-Si <100> with a resistivity of 1–5 Ω·cm. After RCA cleaning, the wafers were stripped of the native oxide by dipping the substrates for 1 min in a 2% HF solution. The wafers were then loaded into a two-chamber PECVD reactor (Elettrorava s.p.a.) where ~5 nm-thick intrinsic a-Si:H layers were deposited on both sides to ensure a good surface passivation. The back side of the wafer was then covered with a ~20 nm n-type a-Si:H film to form the back surface field and electron-selective contact, whereas the front hole-selective contact was realized with the deposition of a ~10 nm p-type a-Si:H layer. Following this, an 80 nm-thick ITO layer was sputtered on the front side through 3.5 cm^2^ shadow masks from a ceramic target with a nominal composition of In_2_O_3_:SnO_2_ (90/10 wt.%), using an Ar atmosphere and powered by DC (Univex 450B, Leybold). At this point, the graphene monolayers were transferred onto the ITO surface. For the current research, we chose to test the effect of adding between one and three monolayers. Finally, after graphene deposition, Ti and Ag metal contacts were evaporated on the front (through a grid) and on the back (full area) and the cells were annealed for 5 min at 200 °C in a hot plate to recover the passivation lost during the ITO sputtering process [9]. A sketch of the finished solar cells is depicted in Figure 1.

The graphene films were characterized as already in place in unfinished solar cells prior to metallization. Raman microscopy using a 514 nm Ar laser (inVia Renishaw) was used to check the quality of the films and to corroborate the number of graphene films transferred [10]. The effect of adding the graphene monolayers on the reflectivity of the solar cells was evaluated using a UV/Visible/NIR Perkin-Elmer Lambda 1050 spectrophotometer. Lastly, the sheet resistance of the hybrid transparent conductive electrodes was evaluated using the noncontact and nondestructive commercial Onyx system from das-Nano Company (see [11] for further details), based on reflection-mode terahertz time-domain spectroscopy (THz-TDS) [12,13].

Finished solar cells were characterized by measuring illuminated current–voltage characteristics at AM1.5G conditions and 100 mW/cm^2^ using a class-A solar simulator (Steuernagel SC575) and external quantum efficiency (EQE). Additionally, Suns-Voc measurements were performed using the Sinton Instrument WCT-120 with the appropriate accessory stage to evaluate the series resistance (*R*_s_) [14].

## 3. Results

In the following subsections, the quality of the graphene films as well as their effect on the TCE optical and electrical performance is presented. Lastly, the results of studies on solar cells where the top ITO has been replaced with the hybrid TCEs are also shown.

### 3.1. Graphene Quality

**Figure 2:**
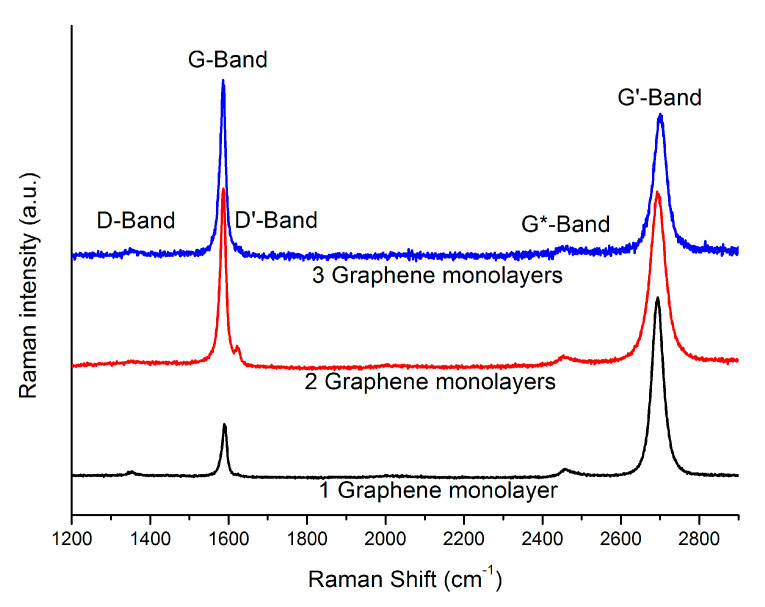
Raman spectra of the graphene layers transferred onto the ITO. The *I*_G_/*I*_G’_ ratio of the monolayer, bi-layer and tri-layer samples lies within the expected range, indicating a successful layer-by-layer transfer process.

Raman spectra of the graphene films already transferred onto the ITO in the solar cells were used to evaluate the quality of the graphene films and transferring process. Three different spectra taken at random points in the cells with one, two and three graphene layers are displayed in Figure 2. All of the spectra show the characteristic peaks expected from graphene layers [15], mainly the G, G* and G’ (or 2D as is also found in the literature) bands appearing at ~1590 cm^−1^, ~2450 cm^−1^ and ~2690 cm^−1^, respectively. In Raman spectra of multilayered graphene stacks, the intensity ratio *I*_G’_/*I*_G_ is dependent on the number of graphene layers [10,15,16]. Experimentally, the intensity ratio typically exhibits a value of *I*_G’_/*I*_G_ > 2 for monolayers, 1 < *I*_G’_/*I*_G_ < 2 for bi-layers and *I*_G’_/*I*_G_ < 1 for tri-layers and beyond. From the spectra shown in Figure 2, the intensity ratios for the samples with monolayers, bi-layers and tri-layers are 3.48 ± 0.20, 0.99 ± 0.02 and 0.82 ± 0.03, respectively (the errors are deduced from the fitting accuracy), which is consistent with the number of layers. This shows that the transferring process does not seem to widely introduce wrinkles or creases in the films otherwise the *I*_G’_/*I*_G_ intensity ratio would not agree with the number of layers transferred [5].

In addition to the G, G’ and G* bands, a rather weak signal is also detected around 1350 cm^−1^ and 1620 cm^−1^, corresponding to the D and D’ bands. The presence of the D and D’ bands require the existence of sp^3^-C defects for their activation. In the spectra shown, the contribution of both peaks is very small and the defects are possibly rather localized, since not all of the spectra taken displayed these peaks. Nevertheless, some degree of imperfection seems to be present in the transferred layers though it is likely not high enough as to have a negative effect in the proposed application.

### 3.2. Hybrid TCE Optical Performance

**Figure 3:**
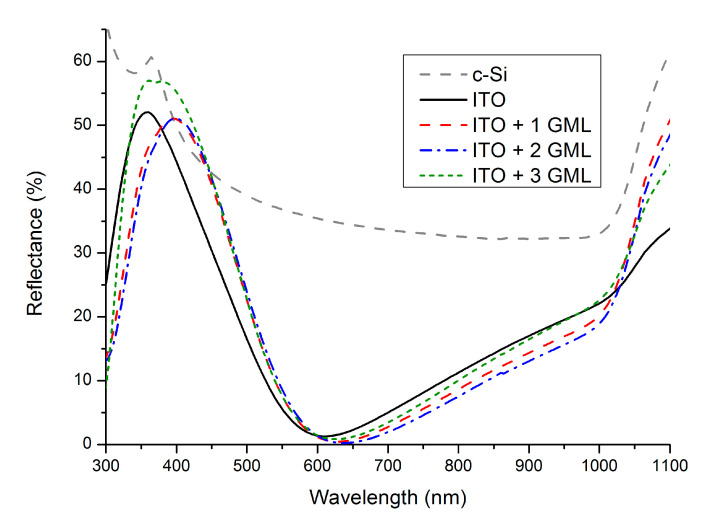
Reflectance spectra measured for the cells with only ITO as the TCE and the cells with a hybrid TCE consisting of a layer of ITO and either one, two, or three graphene monolayers. The reflectance spectrum of a bare polished silicon wafer is also shown for reference.

The addition of graphene monolayers onto the ITO surface modifies the optical characteristics of the transparent conductive electrode. The most straightforward effect is the loss in optical transmittance, which has been found to be about 3% on average with each additional graphene sheet in the wavelength of interest for SHJ solar cells [17]. In the present research, since all of the experiments have been performed at different steps during the fabrication of the solar cells, we have not measured the transmittance of the multilayered stacks. Nevertheless, previous investigations using the same materials deposited onto transparent substrates have validated this [18]. In the case of SHJ solar cells, not only is the transmittance relevant, it is also important to evaluate the reflectivity of the structure since the TCE also serves the role of an antireflective (AR) coating. The reflectance spectra of the structures can be seen in Figure 3.

The spectra obtained are typical for an AR coating deposited on a polished silicon wafer. As can be seen, the presence of ITO is able to greatly reduce the reflectivity of the Si polished wafer (also shown in Figure 3 for reference), especially between 400 nm and 1000 nm. Focusing on the effect of adding one or two graphene monolayers onto the ITO, we can observe how the minimum reflectance shifts towards slightly longer wavelengths. This shift can be understood by reviewing the refractive indexes (*n*) of the films involved. The thickness (*d*) of the ITO films are chosen so that the minimum reflectance occurs at a wavelength *λ*_0_ ≈ 600 nm according to the condition *n·d = λ*_0_/4 [19]. By adding the graphene layers, since the refractive index of graphene in that wavelength range is reported to be around *n* ≈ 2.5 [20], i.e., above that of ITO (*n* ≈ 2), *λ*_0_ has to shift towards higher wavelengths since the thickness remains practically unchanged. In the case of three graphene monolayers, the reflectance spectra measured deviate from the trend created with one and two monolayers and the minimum shifts again towards lower wavelengths.

Besides the shift in the position of the minimum, it is also relevant to assess the AR properties of the TCEs. To do so, the average reflectance weighted by the AM1.5G spectrum (*R*_w_) can be calculated in the wavelength range of interest according to:

(1) $$R_{w}=\frac{\int_{\lambda_{1}}^{\lambda_{2}}R\left(\lambda \right)G_{\mathrm{AM}1.5G}\left(\lambda \right)d\lambda }{\int_{\lambda_{1}}^{\lambda_{2}}G_{\mathrm{AM}1.5G}\left(\lambda \right)d\lambda }$$

From Figure 3, it is clear that the hybrid TCEs provide better AR properties if the wavelength range is limited to 600–1050 nm, whereas outside of that range, ITO mostly has a better performance. However, the majority of the responses of the solar cells studied in this research occur within a much narrower range than that shown in Figure 3. For example, around 95% of the *J*_sc_ of the solar cells is generated within the wavelength range of between 430–1050 nm, as calculated by integrating their external quantum efficiencies (not shown). Within that range, the differences in *R*_w_ for the four structures studied are very small. If the range is narrowed even further to 600–1050 nm, the contribution to *J*_sc_ represents around 80% of the total and, within that range, the hybrid TCEs show up to a 2.8% lower *R*_w_. For clarity, Table 1 gathers the values of *R*_w_ within those particular wavelengths.

### 3.3. Hybrid TCE Electrical Performance

**Figure 4:**
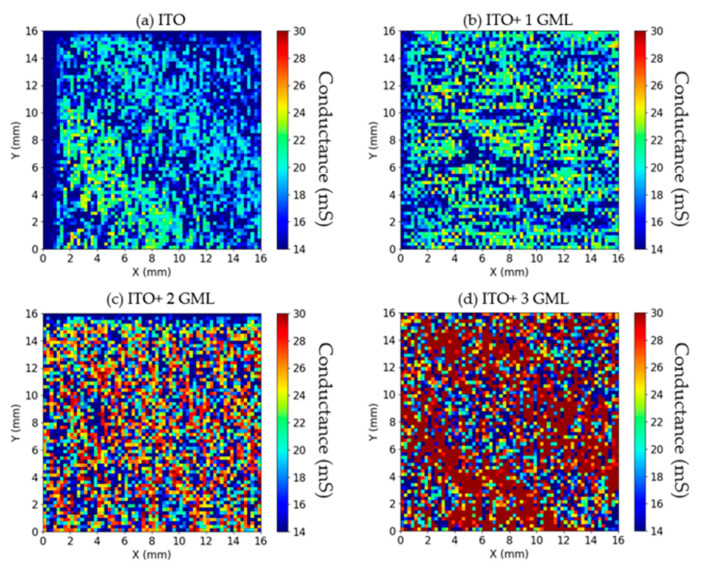
Reflection-mode terahertz time-domain spectroscopy results obtained in the cells with bare ITO (**a**) and with ITO + 1 (**b**), +2 (**c**) and +3 (**d**) graphene monolayers as the front contact TCE.

The main point of interest in introducing graphene into the solar-cell structure is to improve the electrical properties of the transparent conductive electrode (while sacrificing as little as possible of the optical characteristics). In order to characterize the sheet resistance in situ, a contactless technique such as THz-TDS is highly convenient. Figure 4 shows the maps of the conductance obtained for the cells with ITO (a) and with ITO + 1 (b), +2 (c) and +3 (d) graphene monolayers. Undoubtedly, the colormaps show a clear improvement in the TCE conductance as more graphene layers are added.

**Figure 5:**
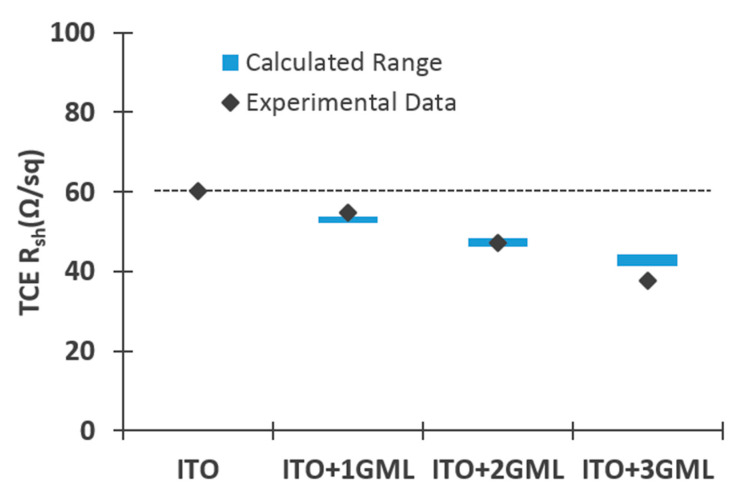
Measured sheet resistance (scatter plot) using THz-TDS and the calculated range of values (blue colored areas) according to Equation (2), considering a sheet resistance for the graphene monolayers of 450 ± 50 Ω/sq. The dotted line is a guide to the line positioned at a value equal to the measured ITO sheet resistance.

When translated to sheet resistance, the conductance maps yield average values that decrease from 60.2 Ω/sq for the cell with bare ITO, to 54.9 Ω/sq, 47.2 Ω/sq, and 37.7 Ω/sq for the cells with one, two, and three graphene monolayers, respectively. Previous results obtained using transmission line measurements [18] revealed a sheet resistance for the ITO of 59 Ω/sq while the graphene layers have a sheet resistance of 450 ± 50 Ω/sq [8]. Since the sheet resistance sees the ITO film and the graphene layers as connected in parallel [21], the obtained values should decrease according to the expression:

(2) $$R_{\mathrm{Sh}}{}^{-1}=R_{\mathrm{Sh}\_\mathrm{ITO}}{}^{-1}+m\cdot R_{\mathrm{Sh}\_\mathrm{Gr}}{}^{-1}$$

where *R*_Sh_ITO_ and *R*_Sh_Gr_ are the sheet resistance of ITO and graphene, and *m* is the number of graphene layers. The values obtained using THz-TDS and the range of expected values considering *R*_Sh_ITO_ = 60.2 Ω/sq and *R*_Sh_Gr_ = 450 ± 50 Ω/sq are plotted in Figure 5. As can be observed, the values obtained match very well to the anticipated values. Therefore, the results indicate that there is good electrical contact between all layers and that the hybrid TCE should outperform bare ITO in terms of the lateral collection of carriers that reaches the TCE.

### 3.4. Solar-Cells Performance

**Figure 6:**
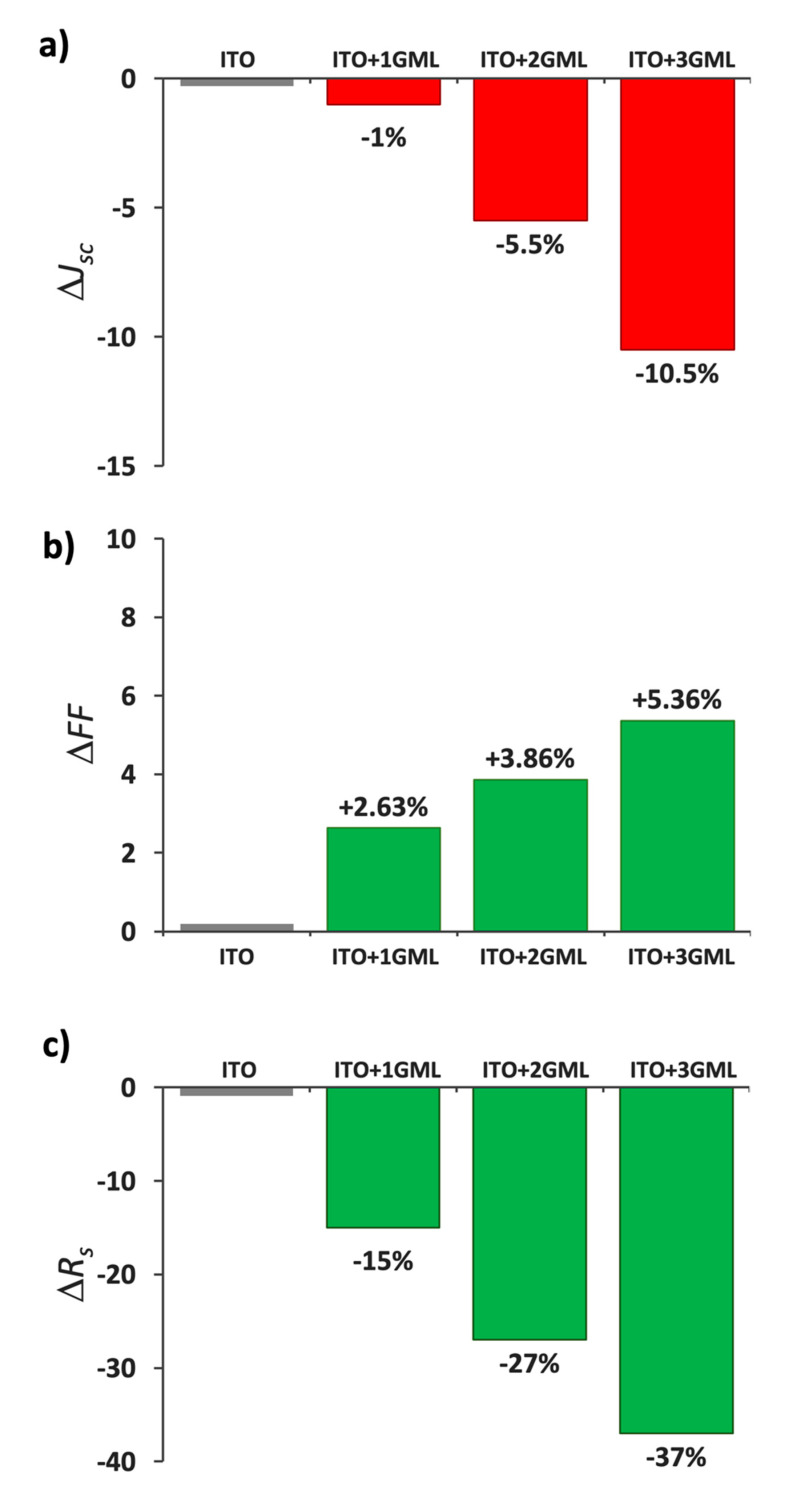

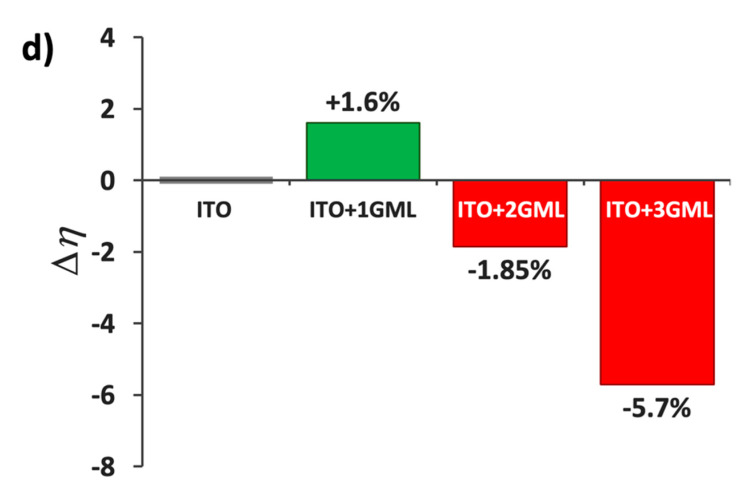
Solar cell parameter variations (*J*_sc_ (**a**), *FF* (**b**), *R*_s_ (**c**) and overall efficiency *η* (**d**)) observed as the front ITO is substituted by a hybrid TCE consisting in an ITO layer covered with one, two or three graphene monolayers.

In reference [18], we reported on the impact of substituting the front ITO in SHJ solar cells by a hybrid TCE consisting of a single graphene monolayer transferred onto the front ITO. We found that the decrease in sheet resistance of the hybrid TCE compared with the bare ITO translated to a lower series resistance, *R*_s_, and an improved *FF*. On the other hand, the overall decrease in transmittance was compensated for by the better AR properties in a large part of the spectrum usable by the solar cells, and *J*_sc_ was hardly affected. Together, the net effect was an improvement in the solar-cell efficiency (*η*). On the basis of these results, for this work we focused on evaluating the effect of going beyond one graphene monolayer. In Figure 6, the changes in *J*_sc_, *FF*, *R*_s_ and the overall efficiency *η* of the cells with hybrid TCEs, with respect to the reference cell with only ITO are presented.

Regarding *V*_oc_, the soft techniques used for transferring the sheets (thus not damaging the underlying layers) and the fact that the sheets are far from the junction means that values should not be altered. Indeed, we found the *V*_oc_ measured in the cell with only ITO (708 mV) remained practically unchanged in the cells with a hybrid TCE (707 mV), and thus are not shown in Figure 6. The values of *J*_sc_ on the other hand saw a strong decrease when more than one graphene monolayer was used, as seen in Figure 6a. Again, with the addition of a single graphene layer, the loss in transmittance is mostly offset by the improvement in reflectance in a large part of the usable light, and only 1% of *J*_sc_ is lost. However, with the addition of a second and third monolayer the loss in transmittance clearly dominates the optical response and the loss in *J*_sc_ worsens to 5.5% and 10.5%, respectively.

Figure 6b,c recapitulate the changes in *FF* and *R*_s_. As shown, the improvements in these cases are noticeable. As the top contact ITO was replaced with a hybrid TCO with one, two or three graphene monolayers, the value of *R*_s_ decreased from 2.46 Ω·cm^2^ down to 2.10 Ω·cm^2^, 1.83 Ω·cm^2^ and 1.65 Ω·cm^2^, respectively; likewise, the *FF* increased from 70.7% to 72.6%, 73.5% and 74.6% accordingly. The observed trend is in line with the improvements in sheet resistance of the TCEs obtained with each additional graphene monolayer shown in Figure 5. In addition, a better workfunction matching between the Ti electrode and the ITO, and thus a lower junction barrier, is also expected thanks to the presence of graphene. The workfunction of Ti is 4.33 eV whereas in ITO it is close to 5.0 eV. According to [5,22], the workfunction of graphene multilayer stacks depends on the number of monolayers, increasing from 4.32 eV for one monolayer, up to 4.55 eV in the case of a three-monolayer stack. Hence, the barrier between Ti and ITO is reduced with each graphene monolayer which would also help in improving the series resistance of the devices [7].

Lastly, the effect on the overall efficiency is summarized in Figure 6d. Since the value of *V*_oc_ remained practically unchanged, the effect of the added graphene monolayers on the efficiency is the result of the compromise between the improved electrical characteristics and the worsening of the optical transparency of the TCE. As a result, the device with a single graphene monolayer displayed a 1.6% higher efficiency compared to the reference cell, but the cells with two and three monolayers suffered an efficiency drop of 1.85% and 5.7%, respectively.

**Figure 7:**
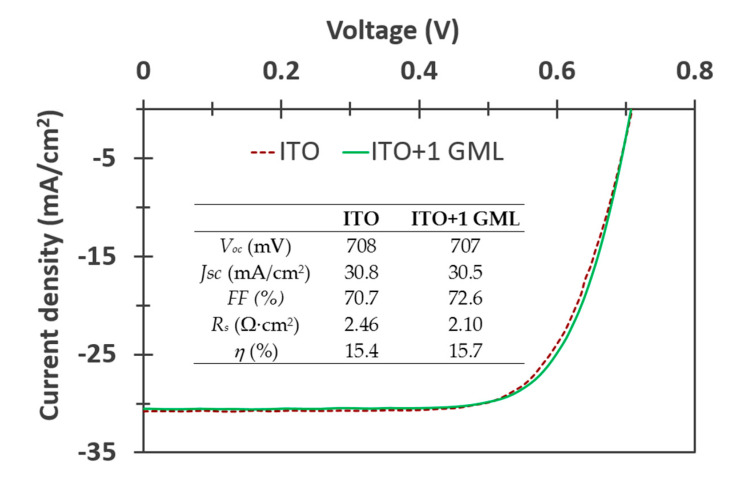
JV characteristics under illumination of the cells with either ITO as the front TCE and with the ITO layer covered with one graphene monolayer.

To conclude, in Figure 7 the current–voltage characteristics under AM1.5G illumination and the extracted parameters for the cells with ITO and with ITO + one GML are shown. These results demonstrate that there is potential in hybrid TCEs based on graphene and transparent conductive oxides. The addition of the graphene monolayers to the ITO clearly improves its electrical characteristics, and the improvements are transferred to the JV characteristics of the solar cells which display a better *R*_s_ and *FF*. The loss in transmission caused with each graphene monolayer means that they need to be kept at a minimum, otherwise the drop in *J*_sc_ can be significant. However, in addition to what we have shown there is still clear room for improvement. Among other things, the electrical conductivity of graphene can be increased via chemical doping without sacrificing its transparency [23]; and the thickness of the TCO can be further optimized for hybrid structures as deduced from the reflectance spectra shown in Figure 3. All these prospects invite further study on hybrid electrodes based on graphene as a promising alternative for next-generation solar cells and compatible upcoming technologies.

## 4. Conclusions

This work presents a study on hybrid transparent conductive electrodes based on ITO and graphene and its application as front contacts in silicon heterojunction solar cells. The electrodes consisted of up to three monolayers of CVD-synthesized graphene transferred onto the top of ITO in silicon heterojunction solar cells.

The obtained Raman spectra indicated the presence of good quality graphene in stacks of one, two and three monolayers. The effect of the graphene layers on the reflectance spectra depended on the wavelength range considered. In the range between 430–1050 nm (which corresponds to around 95% of the generated photocurrent) a small increase in the weighted reflectance not larger than 1.5% was observed. In the range between 600–1050 nm (which corresponds to around 80% of the generated photocurrent) all of the cells with graphene on top showed better antireflectance properties than the reference cell. The electrical characterization through THz time domain spectroscopy illustrated good electrical contact between the ITO and the stacks of graphene monolayers. For all cases, the sheet resistance of the ITO layer was reduced accordingly to an intimate parallel combination of all the layers.

The current–voltage characteristics of the solar cells with graphene showed a better series resistance and *FF* thanks to the improved sheet resistance and workfunction matching between the transparent electrode and the metal grid, while the cells’ *V*_oc_ was hardly affected. On the other hand, *J*_sc_ decreased with each graphene monolayer due to a lower optical transmission of the electrode. This effect was mostly cancelled out for one graphene monolayer by the improvement in reflectance in a large part of the usable light, and only 1% of *J_sc_* was lost. Overall, the cell with a single graphene monolayer displayed a 1.6% higher efficiency compared to the reference cell, while the cells with two and three monolayers could not improve upon that.

These results show that graphene can be used to improve the conductance of ITO and that its application can be helpful in increasing the efficiency of solar cells. Furthermore, new strategies to improve the chemical doping of graphene monolayers without sacrificing its transparency are being studied worldwide. The application of such strategies to hybrid electrodes based on graphene, or even to electrodes based on multilayered graphene alone, opens up the possibility of fabricating high-performance electrodes suitable for use in next-generation solar cells and upcoming technologies.

## Figures and Tables

**Table 1:** Weighted reflectance calculated from the spectra shown in Figure 2 in two different wavelength ranges. The range between 430–1050 nm is relevant because it represents around 95% of the photogenerated current; the range between 600–1050 nm is the range where the hybrid TCE is clearly better in terms of antireflectance properties, and it accounts for around 80% of the photogenerated current.

TCE	*R*_w_ 430–1050 nm (~95% *J*_sc_)	*R*_w_ 600–1050 nm (~80% *J*_sc_)
ITO	12.05%	10.51%
ITO + 1 GML	12.76%	8.65%
ITO + 2 GML	12.56%	7.74%
ITO + 3 GML	13.62%	9.81%
